# Gender-Specific Differences in the Intensive Care Treatment of COVID-19 Patients

**DOI:** 10.3390/jpm12050849

**Published:** 2022-05-23

**Authors:** Peter Jirak, Moritz Mirna, Vincent Van Almsick, Zornitsa Shomanova, Magdalena Mahringer, Michael Lichtenauer, Kristen Kopp, Albert Topf, Franz Sieg, Johannes Kraus, Sarah X. Gharibeh, Uta C. Hoppe, Lukas Fiedler, Robert Larbig, Rudin Pistulli, Lukas J. Motloch, Anna-Maria Dieplinger

**Affiliations:** 1 Clinic II for Internal Medicine, University Hospital Salzburg, Paracelsus Medical University, 5020 Salzburg, Austria; m.mirna@salk.at (M.M.); magdalena.mahringer@stud.pmu.ac.at (M.M.); m.lichtenauer@salk.at (M.L.); k.kopp@salk.at (K.K.); f.sieg@salk.at (F.S.); j.kraus@salk.at (J.K.); s.gharibeh@salk.at (S.X.G.); u.hoppe@salk.at (U.C.H.); lukas.fiedler@wienerneustadt.lknoe.at (L.F.); l.motloch@salk.at (L.J.M.); 2 Department of Cardiology I, Coronary and Peripheral Vascular Disease, Heart Failure, University Hospital Muenster, 48149 Muenster, Germany; vincentfrederic.vanalmsick@ukmuenster.de (V.V.A.); zornitsa.shomanova@ukmuenster.de (Z.S.); rudin.pistulli@ukmuenster.de (R.P.); 3 Clinic for Internal Medicine, Hospital Villach, 9500 Villach, Austria; albert.topf@kabeg.at; 4 Department of Internal Medicine II, Wiener Neustadt Hospital, 2700 Wiener Neustadt, Austria; 5 Division of Cardiology, Hospital Maria Hilf Mönchengladbach, 41063 Mönchengladbach, Germany; robert.larbig@mariahilf.de; 6 Department of Cardiology II-Electrophysiology, University Hospital Muenster, 48149 Muenster, Germany; 7 Nursing Science Programme, Institute for Nursing Science and Practice, Paracelsus Medical University, 5020 Salzburg, Austria; annamaria.dieplinger@ooeg.at; 8 Medical Faculty, Johannes Kepler University Linz, 4040 Linz, Austria

**Keywords:** COVID-19, gender, sex, intensive care, ICU

## Abstract

Background: Gender-specific differences in the outcome of COVID-19 patients requiring intensive care treatment have been reported. However, a potential association with ICU therapy remains elusive. Methods: A total of 224 consecutive patients (63 women) treated for severe COVID-19 disease requiring mechanical ventilation were screened for the study. After propensity score matching for gender, 40 men and 40 women were included in the study. Comparative analysis was conducted for laboratory parameters, ICU therapy and complications (pulmonary embolism, thrombosis, stroke, and ventricular arrhythmias), and outcome (mortality). Results: Male patients had significantly higher levels of CRP (*p* = 0.012), interleukin-6 (*p* = 0.020) and creatinine (*p* = 0.027), while pH levels (*p* = 0.014) were significantly lower compared to females. Male patients had longer intubation times (*p* = 0.017), longer ICU stays (*p* = 0.022) and higher rates of catecholamine dependence (*p* = 0.037). Outcome, complications and ICU therapy did not differ significantly between both groups. Conclusion: The present study represents the first matched comparison of male and female COVID-19 patients requiring intensive care treatment. After propensity matching, male patients still displayed a higher disease severity. This was reflected in higher rates of vasopressors, duration of ICU stay and duration of intubation. In contrast, no significant differences were observed in mortality rates, organ replacement therapy and complications during ICU stay.

## 1. Introduction

The COVID-19 pandemic, triggered by SARS-CoV-2 infection, represents an ongoing challenge for health-care systems to date [1]. SARS-CoV-2 represents a single-stranded RNA virus of the coronavirus family [2]. While the severity of the disease displays a broad range from asymptomatic or mild courses to severe pneumonia and acute respiratory distress syndrome (ARDS), bilateral pneumonia, hypoxaemia and respiratory failure represent the most typical manifestations of the disease [3,4]. Still, severity is also dependent on the virus variant [5]. The binding of SARS-CoV-2 to the spike protein S represents a key factor in the pathogenesis of COVID-19 [2,6]. Through the binding of SARS-CoV-2 to the spike domain of the ACE-2 receptor on the cell surface, the virus is subsequently internalised and replicated by the host cell [7]. As a result, organs with a high expression of the ACE-2 receptor are particularly susceptible to be affected by the virus [7,8]. Furthermore, in the context of ACE-2-mediated virus uptake, spike protein priming of SARS-CoV-2 by transmembrane protease serine 2 (TMPPRSS2) plays an essential role [9]. Through cleavage of the ACE2 tail, TMPRSS2 facilitates the SARS-CoV-2 passage and leads to an increase in the viral load [10].

A frequent complication of COVID-19 disease is an excessive inflammatory reaction, resulting in a cytokine storm and a dysregulation of inflammatory processes [11,12,13,14,15]. However, excessive inflammatory reaction can occur in the majority of bacterial and viral infections and does not represent a COVID-19-specific reaction [14]. Given the role of the ACE-2 receptor in virus internalization, along with the in part excessive inflammatory response, COVID-19 disease can lead to the damage of virtually all organ systems, similar to other septic conditions [13,16]. Numerous risk factors have already been identified for a severe and fatal disease course. Interestingly, among classical risk factors, such as old age, cardiovascular disease, diabetes, lung disease and the presence of autoimmune diseases or immunodeficiency [15,17], gender-specific differences have also been reported. An early study by Jin et al. observed a higher risk for a severe disease course and mortality in men, despite a similar disease prevalence [18]. This was confirmed in an analysis of COVID-19-related deaths, which reported a 1.77 times higher mortality rate for male patients [19]. Of note, this effect was observed worldwide and thus seems to be independent of potential geographic influences [20]. Various explanations have been discussed as potential reasons for this observation. On a molecular basis, higher expressions of ACE-2, as well as TMPPRSS2, occur in male patients, which are both key factors for cell entrance of the virus [9,20]. Moreover, differences in the immune response in males and females have to be considered [21]. However, potential differences in the risk profile for a severe and fatal disease course between male and female patients also have to be taken into account [15,20,22]. This is further emphasized by sex-specific differences in risk factors for other disease entities, such as cardiovascular disease [6]. To date, it remains unclear whether differences in medical care and treatment itself might represent a further cause for the aforementioned differences in mortality. In this regard, intensive care treatment represents a key factor indicative of further disease course. Accordingly, the present study aims to clarify whether gender-specific differences in treatment at the ICU, independent of further risk factors, might affect the clinical outcome of COVID-19 patients requiring intensive care treatment [23]. We hypothesized that sex-specific differences in intensive care treatment might represent a potential cause for worse outcomes observed in male COVID-19 patients.

## 2. Materials and Methods

The present study was conducted in accordance with the standards of good clinical practice and the principles of the Declaration of Helsinki and was carried out in three tertiary centres in Germany and Austria. The study was approved by the respective local ethic committees (University Hospital Münster Nr. 2020-306-f-S, Maria Hilf Hospital Mönchengladbach: Nr. 143/2020 and University Hospital Salzburg: Nr. 1071/2020).

### 2.1. Study Cohorts

In total, 224 consecutive patients (63 women) who were treated at an intensive care unit due to severe COVID-19 disease requiring mechanical ventilation (defined as requirement for high-flow nasal cannula, non-invasive and/or invasive ventilation) between 4/2020 and 1/2021 were screened for the present study. After propensity score matching for sex, 40 men and 40 women were included in the study. The diagnosis of COVID-19 was made on the basis of a positive result of an oropharyngeal or/and nasopharyngeal swab test by real-time reverse transcription–polymerase chain reaction assay for COVID-19 (performed according to the manufacturer). Radiographic confirmation by means of a chest radiography and/or computer tomography of the thorax was required for the diagnosis of COVID-19 or COVID-19-related pneumonia. Intensive care treatment was completed in all patients included in the study, meaning they either were discharged or had died at the time of data analysis. In the study, variables were classified according to the Input/Throughput/Output and the Outcome model of Pfaff was applied [24].

### 2.2. Data Collection

For all patients screened for the study, demographics, medical history, laboratory examinations, comorbidities, complications, specific treatment measures and outcomes during intensive care treatment were captured in the respective hospital database and analysed retrospectively.

### 2.3. Statistical Analysis

The statistical analysis was performed by a blinded statistical analytic team using SPSS (Version 23.0, IBM, Armonk, NY, USA) and R (version 4.0.2., R Core Team (2013), R Foundation for Statistical Computing, Vienna, Austria; http://www.R-project.org/) with the packages ‘Rcmdr’, ‘ggplot2’, ‘pastecs’, ‘Hmisc’, ‘ggm’, ‘polycor’, ‘QuantPsyc’, ‘glmnet’, ‘Matching’, ‘MatchIt’, ‘optmatch’, ‘RItools’, ‘Rcpp’, ‘stddidff’ and ‘jtools’. The distribution of continuous data was assessed visually and by applying a Kolmogorov–Smirnov test. Because data were not normally distributed, medians ± interquartile range (IQR) were depicted and medians were compared using Mann–Whitney-U test. Categorical data were analysed using Fisher’s exact test. Propensity score matching was conducted to account for covariate imbalances with a possible impact upon outcome. Standardized differences between the two groups were calculated. Covariates with statistically significant differences between groups or standardized differences >0.25 were included in the matching. The ‘nearest neighbor’ matching approach with a 1:1 ratio and a calliper of 0.25 was used for propensity score matching. A *p*-value of <0.05 was considered statistically significant.

## 3. Results

### 3.1. Baseline Characteristics

The baseline characteristics of enrolled patients are depicted in Table 1. In total, 224 patients were enrolled in this study, of whom 63 patients were female (28.1%). Coronary artery disease was significantly more prevalent in male patients (23.0% vs. 7.9%, *p* = 0.012), whereas history of thrombosis was more prevalent among females (6.2% vs. 15.9%, *p* = 0.023).

### 3.2. Propensity Score Matching

**Figure 1:**
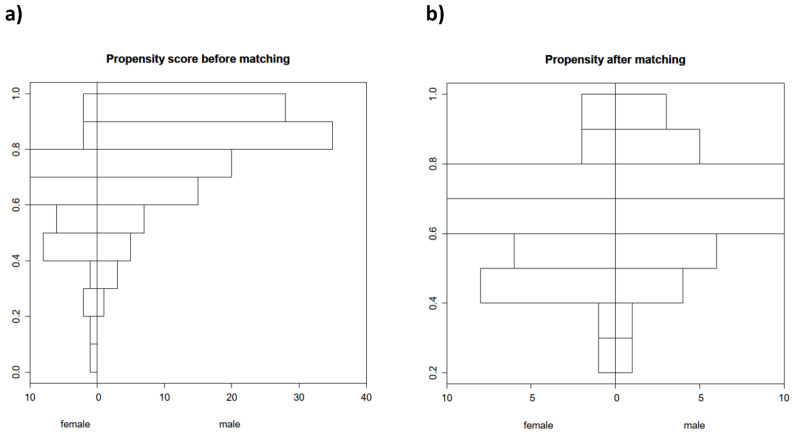
Distribution of propensity scores (**a**) before (*n* = 224) and (**b**) after propensity score matching (*n* = 80).

**Figure 2:**
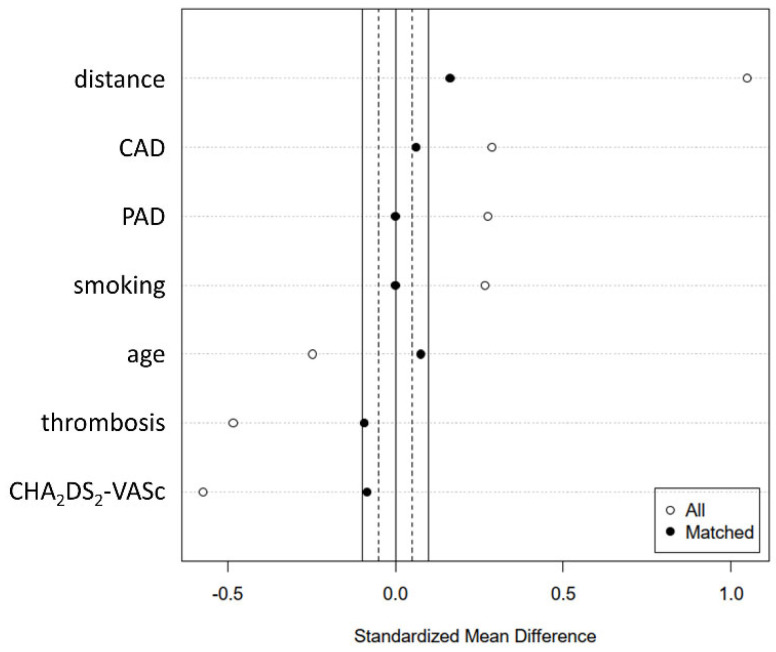
Standardized mean differences in included covariates before and after propensity score matching.

Propensity score matching was performed to account for covariate imbalances between the two investigated groups. Covariates with statistically significant differences in frequencies or medians and/or standardized differences >0.25 (age, CHA2DS2-VASc score, coronary artery disease, peripheral artery disease, history of smoking, and history of thrombosis) were included (see Figure 1 and Figure 2).

### 3.3. Characteristics of Matched Groups-Input

After propensity score matching, there were no statistically significant differences in any of the aforementioned comorbidities (also see Table 1) between the two matched groups. The laboratory parameters of matched groups are depicted in Table 2. Of note, minimum pH was lower in matched males (median 7.20 (IQR 7.13–7.32) vs. 7.31 (IQR 7.15–7.40), *p* = 0.014), whereas serum creatinine (median 1.61 mg/dL (IQR 1.11–3.50) vs. 1.10 mg/dL (IQR 0.84–2.13), *p* = 0.027), C-reactive protein (CRP; median 26.7 mg/dL (IQR 16.13–32.6) vs. 18.6 mg/dL (IQR 11.9–26.8), *p* = 0.012) and interleukin 6 (median 436 pg/mL (IQR 160–1347) vs. 161 pg/mL (IQR 61–545), *p* = 0.020, see Table 2) were significantly higher when compared to matched females.

### 3.4. Therapy at the ICU—Output

After propensity score matching, therapy-specific parameters and outcome parameters were investigated. Notably, matched males had significantly longer ICU stays (median 13 days (IQR 6–25) vs. 10 days (IQR 5–15), *p* = 0.022) and a longer duration of intubation (median 12 days (IQR 4–18) vs. 6 days (IQR 0–11), *p* = 0.017) than matched females. Furthermore, a significant difference was observed between the two groups in the administration of catecholamines, which were given more often in males than in females (87.2% vs. 67.5%, *p* = 0.037; see Table 3).

### 3.5. Complications—Outcome

There were no statistically significant differences in outcome parameters between patients of the two matched groups. However, although not statistically significant, a trend towards higher mortality and ventricular arrhythmias was observed in matched males when compared to females (see Table 4). Overall mortality in our study cohort was 46.25%.

## 4. Discussion

Despite the similar disease prevalence, a higher risk for severe disease and mortality in males was reported compared to females in recent studies [18,19]. Still, the reasons for these findings are not fully understood. To further assess a potential correlation of gender-specific outcomes with differences in medical care and treatment, our study aimed to assess treatment differences in male and female patients requiring intensive care treatment for COVID-19 pneumonia, with a focus on output and outcome [24].

Similar to the findings of previous studies, coronary artery disease was more prevalent in male patients in the unmatched cohort [25]. Consequently, a higher cardiovascular risk in male patients might also result in worse outcomes in COVID-19, as has been recently proposed in the literature [17]. On the other hand, female patients in the unmatched cohort had higher rates of thromboembolic events in their medical histories. This finding is also in line with former studies, reporting an up to two-fold higher risk for venous thromboembolisms in women of reproductive age infected with the SARS-CoV-2 virus [26]. To note, hormonal contraceptives and pregnancy represent a prominent trigger for VTEs in this respect [26]. However, interestingly, an actually higher intrinsic risk of VTE was reported for men compared to women [26].

Based on the findings of earlier studies, propensity matching according to baseline characteristics and risk factors for severe COVID-19 disease was conducted to provide comparability and avoid a potential influence of potential imbalances in risk profiles on our results. To account for the high rates of thromboembolic events reported in severe COVID-19, as well as the gender-specific differences in thromboembolic risk, the CHA2DS2-VASc score was included in the matching criteria. To the best of our knowledge, this study represents the first matched comparison analysis of gender-specific differences in COVID-19 patients requiring intensive care treatment.

With respect to mortality rates, death was more frequent in male patients, yet no statistical significance was observed. However, the comparably small sample size along with the high overall mortality must be considered when interpreting these results. Although mortality rates were high, the overall mortality observed in our study is in line with former studies conducted during a comparable observation period [27]. It should be noted that mortality rates only cover the duration of the ICU stay. Potential gender-specific differences in medium- and long-term outcomes therefore are not reflected in our study.

With respect to myocardial injury, represented by an elevation in maximum high-sensitive troponin levels, no significant differences were observed between groups, while again a trend towards higher levels in male patients was evident. Furthermore, male patients displayed significantly higher creatinine levels, indicating worse renal function compared to female patients. Similarly, elevated levels of CRP and interleukin-6 point towards an increased inflammatory activity in male patients. Based on these findings, a higher disease severity in male patients may be assumed, also in the context of matched risk factors and baseline characteristics. This assumption is further supported by significantly lower pH levels occurring in male patients, again suggesting a higher disease severity. In this regard, the higher expression of ACE-2, as well as TMPPRSS2, in male patients might offer a potential explanation for our findings, as both ACE-2 and TMPPRSS2 are involved in the processes of cell entrance by SARS-CoV-2 [9,20]. Thus, a higher receptor expression might consequently promote a higher disease severity. On the other hand, male and female patients display differences in their immune response pattern, again with higher inflammatory burden and disease severity occurring in male patients [28]. Correspondingly, male patients had significantly longer mechanical ventilation times, as well as higher rates of catecholamine dependence and a significantly longer overall stay in the ICU. These findings again indicate a potentially higher disease severity in male patients. While a higher disease severity might contribute to the worse outcomes in male patients, this was not fully reflected by the need for specific ICU treatment regimes. No differences in the rates of COVID-19-specific medical therapy were observed between both groups, either in the use of corticosteroids, remdesivir, tacilizumab or in the use of therapeutic anticoagulation. Of note, corticosteroids were given to all patients in our study collective, based on the results of the “Recovery-Trial” [29]. Accordingly, no differences between the two groups were expected in this regard. However, this does not account for the similar rates of remdesivir, tacilizumab, as well as therapeutic anticoagulation. Similarly, rates of ECMO, hemofiltration, as well as intubation, did not differ between male and female patients and no significant differences with respect to the rates of complications at the ICU were observed in our study. However, it has to be pointed out that this might also be due to the relatively small sample size of our study, resulting in a low overall event rate in our study collective.

Gender-specific differences have already been subject to ICU research prior to the COVID-19 era. In this regard, the “Frog-ICU” study represents one of the most important studies on the impact of gender on survival in critically ill patients [30]. Interestingly, similar to our study, women constituted only one-third of the study population, while a similar survival rate after ICU admission was evident for males and females, independent of comorbidities and disease severity [30]. Similarly, the duration of ICU stay (12 days) was comparable to our study results, while overall mortality rates were considerably lower (21.7%) [30]. Of note, a higher admission number of males compared to females, along with a higher use of resources, have also been reported in a recent study by Lat et al. [31]. Additionally, with regard to ICU treatment, a longer time to antibiotic initiation, less dialysis, as well as less mechanical ventilation and less initiation of vasopressors, have been reported for female patients in ICU treatment [32,33,34]. However, no propensity matching was applied in these studies. In comparison, the gender differences in our study seem to be attenuated, potentially also promoted by matching of the study cohorts. Based on the higher disease severity discussed above, the differences observed in ICU therapy in our study can be considered plausible findings and do not necessarily point towards gender differences in intensive care treatment. While one might expect further differences in ICU therapy with a rise in disease severity, the comparable rates of organ replacement therapy in both groups also point against potential differences in treatment. However, further interpretation in this context is also limited by the sample size in our study.

While no significant differences were observed with regard to outcomes, in our study, the higher disease severity, along with longer intubation times and ICU stays in male patients, still represent findings of concern from a public health perspective. On the one hand, potentially long-term effects due to longer intubation times and ICU stays can be assumed, leading to prolonged rehabilitation periods and potentially worse long-term outcomes [35,36,37]. This again might cause a reduction in the working capacity of the respective patients, resulting in a negative economic effect [35,37]. On the other hand, these findings also accompany an increase in treatment costs. Additionally, considering the shortage in the number of intensive care beds during the peaks of the COVID-19 pandemic, the significantly higher duration of ICU stays might further add to this problem [38,39]. This might ultimately promote the overstrain of hospitals, with a decrease in the quality of medical care and, in the worst case, triage of ICU and ventilation capacities [38,40]. Thus, the need for preventive strategies and awareness, especially in male patients, with respect to severe COVID-19 is emphasized by our findings. This is of special importance, considering the commonly described lower health awareness in male patients compared to females in the literature [41].

## 5. Conclusions

The present study represents the first matched comparison of male and female COVID-19 patients requiring intensive care treatment. After propensity matching for baseline characteristics and risk factors for COVID-19, male patients still displayed a higher disease severity. This was reflected in higher rates of vasopressors, duration of ICU stay and duration of intubation. In contrast, no significant differences were observed in mortality rates, organ replacement therapy and complications during ICU stay between the two matched groups.

## 6. Limitations

While contributing novel clinical findings, the present study has by design some limitations. First, the analysis was conducted retrospectively. Moreover, due to the small sample size, our findings have to be considered as hypothesis-generating. Additionally, with respect to overall event rates of ICU complications, the sample size has to be considered a major limiting factor. Similarly, no follow-up analyses were conducted. Thus, potential medium- and long-term effects after ICU discharge are not covered by our study. Moreover, data on bacterial superinfections were not included in the analysis due to limited data availability. Since our data were investigated in severe COVID-19 requiring intensive care treatment and mechanical ventilation, our results cannot be applied in less severe COVID-19 clinical scenarios. No mutation analysis was available for the patients included in our study. Still, with regard to a potential interference of virus mutations with our findings, the first SARS-CoV-2 variants, B.1.1.7 and B.1.351, were declared a variant of concern on 18th of December 2020, followed by P.1 on 11th of January 2021 [42]. Keeping in mind the observation period of our study, which only extended up to 01/2021, no relevant impact on our results due to virus mutations is assumed.

## Figures and Tables

**Table 1:** Baseline characteristics.

	Male (*n* = 161)		Female (*n* = 63)		*p*-Value	Std. Diff.
	Median	IQR	Median	IQR		
Age (years)	68	65–79	64	58–78	0.212	0.27
CHA_2_DS_2_-VASc score	2	2–4	3	2–4	0.548	0.58
BMI (kg/m^2^)	27.7	25.3–30.4	28.5	25.3–35.1	0.315	0.13
	%	*n*	%	*n*	*p*-value	
Coronary artery disease	23.0	37	7.9	5	0.012 *	0.33
Diabetes mellitus	33.5	54	34.9	22	0.876	0.01
Arterial hypertension	61.5	99	66.7	42	0.539	0.08
History of smoking	27.3	44	20.6	13	0.314	0.30
Peripheral artery disease	7.5	12	1.6	1	0.117	0.39
Heart failure	11.8	19	12.7	8	0.990	0.11
Valvular heart disease	8.1	13	6.3	4	0.785	0.15
Obstructive pulmonary disease	18.0	29	25.4	16	0.265	0.11
Structural pulmonary disease	5.6	9	11.1	7	0.247	0.07
Pulmonary arterial hypertension	3.1	5	3.2	2	0.990	0.11
Atrial fibrillation	15.5	25	15.9	10	0.949	0.03
History of stroke	6.2	10	6.3	4	0.969	0.15
History of thrombosis	6.2	10	15.9	10	0.023 *	0.38
Malignancy	8.7	14	7.9	5	0.855	0.17

* *p* < 0.05.

**Table 2:** Laboratory parameters of both groups after propensity score matching.

	Male (*n* = 40)		Female (*n* = 40)		*p*-Value
	Median	IQR	Median	IQR	
Age (years)	64	58–78	68	65–79	0.212
CHA_2_DS_2_-VASc score	2	2–4	3	2–4	0.548
BMI (kg/m^2^)	27.7	25.3–30.4	28.5	25.3–35.1	0.315
CK max (U/L)	321	172–731	323	124–567	0.339
CK-MB max (U/L)	25	19–38	30	23–44	0.302
High-sensitivity troponine max (% ULN)	336	164–789	183	101–494	0.091
pBNP max (pg/mL)	976	355–4280	1189	546–4255	0.439
Lactate max (U/L)	2.67	2.12–4.00	3.05	2.18–4.74	0.617
pH min	7.20	7.13–7.32	7.31	7.15–7.40	0.014 *
Creatinine max (mg/dL)	1.61	1.11–3.50	1.10	0.84–2.13	0.027 *
Potassium min (mmol/L)	3.4	3.3–3.6	3.3	3.2–3.8	0.480
Leukocyte count max (G/L)	14.0	11.4–23.1	18.8	11.6–25.5	0.169
Lymphocyte min (G/L)	3.9	0.8–7.3	4.3	1.0–9.2	0.347
CRP max (ng/mL)	26.7	16.13–32.6	18.6	11.9–26.8	0.012 *
PCT max (ng/mL)	1.00	0.59–2.90	0.69	0.22–1.97	0.152
Interleukin 6 max (pg/mL)	436	160–1347	161	61–545	0.020 *

* *p* < 0.05.

**Table 3:** Therapy-specific parameters during ICU stay between the two matched groups.

	Male (*n* = 40)		Female (*n* = 40)		*p*-Value
	Median	IQR	Median	IQR	
Duration of corticosteroids (days)	8	0–10	9	0–10	0.879
Stay on ICU (days)	13	6–25	10	5–15	0.022 *
Time intubated (days)	12	4–18	6	0–11	0.017 *
	%	*n*	%	*n*	*p*-value
Corticosteroids	62.5	25	67.5	27	0.815
Remdesivir	10.0	4	17.5	7	0.518
Tacilizumab	15.0	6	7.7	3	0.481
Therapeutic anticoagulation	67.5	27	77.5	31	0.453
Catecholamines	87.2	34	67.5	27	0.037 *
Intubation	85.0	34	75.0	30	0.402
ECMO	20.0	8	17.5	7	0.775
Hemofiltration	22.5	9	12.5	5	0.378

* *p* < 0.05.

**Table 4:** Complications during ICU stay between the two matched groups.

	Male (*n* = 40)		Female (*n* = 40)		*p*-Value
	%	*n*	%	*n*	
Death	56.8	21	40.0	16	0.174
Pulmonary embolism	0.0	0	10.0	4	0.116
Thrombosis	5.0	2	5.0	2	0.990
Stroke	2.5	1	2.5	1	0.990
Ventricular arrhythmias	10.3	4	5.0	2	0.432

## Data Availability

All presented data including identified study participant data are available upon reasonable request to the corresponding author: Peter Jirak at contact: p.jirak@salk.at. Reuse only permitted after agreement of all co-authors of this study.
